# NIOSH Course for Nurses on Workplace Violence

**Published:** 2013-08-30

A free online course has been created to train nurses on recognizing and preventing workplace violence. The National Institute for Occupational Safety and Health (NIOSH) worked with health-care stakeholders, including nursing and labor organizations, academic groups, and other government agencies, to develop the course. The multimedia training incorporates lesson text, videos depicting workplace violence incidents, personal experiences of nurses with violence on the job, and lesson quizzes. Nurses can receive continuing education credits for completing the online course.

The course is separated into 13 units, each expected to take approximately 15 minutes to complete. Participants can restart the course where they last left off, allowing them to manage their time for taking the course. The course is available on the NIOSH website at http://www.cdc.gov/niosh/topics/violence/training_nurses.html.

